# Editorial: New Educational Technologies and Their Impact on Students' Well-Being and Inclusion Process

**DOI:** 10.3389/fpsyg.2021.753471

**Published:** 2021-11-25

**Authors:** Mirta Vernice, Barbara Carretti, Daniela Sarti, Daniela Traficante, Maria Luisa Lorusso

**Affiliations:** ^1^Department of Humanities, University of Urbino Carlo Bo, Urbino, Italy; ^2^Department of General Psychology, University of Padua, Padua, Italy; ^3^Fondazione Istituto Ricovero Cura a Carattere Scientifico (IRCCS) Istituto Neurologico Carlo Besta, Milan, Italy; ^4^Department of Psychology, Catholic University of the Sacred Heart, Milan, Italy; ^5^Eugenio Medea (Istituto Ricovero Cura a Carattere Scientifico), Bosisio Parini, Italy

**Keywords:** innovative technology in education, inclusion, students well-being, technology and learning, teaching efficacy

The Research Topic collects contributions that allow to rethink educational goals in the digital era from new angles of exploration. Moving from the assumption that introducing innovative technology paradigms in education requires unraveling aspects related to the impact of digital technologies on students' well-being, teaching efficacy and learning success, the Research Topic addressed these points by proposing novel tools and didactic methodologies to be implemented in the educational practices. These aspects appear even more relevant after the pandemic breakout that forced the educational systems of most countries to rely on digital education mainly.

The first point addressed in the Research Topic highlights the impact new technologies play on students' well-being. In this vein, Mascia et al. shed light on the relationship between dysfunctional use of smartphones and adolescent well-being, identifying two dimensions that can affect their quality of life (QoL), emotional intelligence and self-regulation. The authors observed a potential moderating effect of smartphone addiction on the relation between self-regulation and well-being and between emotional intelligence and well-being. Using a performance-based test and self-reports, Sarti et al. explored whether general well-being and school engagement of students with Specific Learning Disabilities (SLD) and Typical Development (TD) could be related to emotional and socio-cognitive functioning. The authors included a Smartphone addiction scale to examine its link with emotional and social functioning. Crucially, students with SLD represent a population vulnerable to problematic smartphone use, as they reported more internalizing and externalizing problems and more difficulties in reaching satisfying and supportive relationships. Importantly, digital well-being concerns the supposed benefits of digital engagement and some of its possible risks; in this view, the digital well-being of students may differ substantially from that of educational staff. The work by Panesi et al. involved the use of the SELFIE platform (a self-assessment tool) that offered a holistic view of how students, teachers, and school leaders perceive the digital *status quo* of their policies and practices, encompassing key aspects in education contexts like students' inclusion.

Second, other studies addressed the potential contribution of ICT to cognitive and neuropsychological assessment by presenting online tools developed to test online verbal comprehension (Caccia et al.), sentence comprehension (Vernice et al.), text reading comprehension (Capodieci et al.), and executive functions (Berg et al.). The need for such tools has been highlighted in the recent pandemic, where the use of online testing and training tools has become a necessity more than a choice. Clinicians and teachers have explored all the possible adjustments of traditional activities to make them fit online use, and a sort of huge natural experiment has taken place highlighting the advantages and disadvantages of the different delivery modalities. Some of the difficulties that children and students may face when reading and processing online information have been analyzed in the study by Caccia et al. in this study, online comprehension is at the same time the object and the means of investigation. On the other hand, the study by Vernice et al. shows how the transition from traditional testing to online testing allows for valuable, large-scale information to be collected that can provide not only data on individual skills but also in-depth analysis of the relationships existing among environmental factors and neuropsychological functioning. Finally, as shown by Capodieci et al.'s study (Capodieci et al.), the growing diffusion of technology in many fields of school life, along with the use of an increasing number of digital reading devices, offers new opportunities to improve traditional reading comprehension and learning skills, extending to more general cognitive abilities such as, for instance, inference generation. In this perspective, internet-based rehabilitation activities may constitute a valid alternative to more traditional, in-presence treatment for learning disorders. Berg et al.'s work (Berg et al.) complete this overview of applications and ICT tools, showing that neuropsychological assessment, in this case specifically of executive functions, in the form of games, can successfully be transferred to non-clinical settings and young, primary school children. This study highlights the importance of finding an ideal (and often hard to determine) level of difficulty that may keep the proposed digital activities challenging but still enjoyable, and proposing a sufficiently varied set of items and trials, but carefully avoiding the risk for the test to become too long and demanding.

A third point emphasized to what extent technology may promote a committed learning environment. In this regard, Feraco et al. explored the effect of the use of interactive teaching practices on academic performance in large classes where attendance is not compulsory. In particular, they focused their attention on the so-called Student Response Systems (SRS) which require the student to answer quizzes during university lessons. The use of quizzes has been integrated with extra-curricular activities that require an in-depth study of the course contents, such as laboratory experiences and writing reports. Both activities were successful in improving the students' final exam. However, these extra-curricular experiences had a positive effect not only on the students' academic performance but also on their motivation. This was not the case with quizzes. To better understand the relationship between technology, motivation, and learning outcomes, An et al. studied this issue in the context of language learning. They found that students with a higher level of motivation (in terms of self-efficacy) experienced greater involvement in activities that promote effective language learning, such as those equipped with technological tools, with better results on English learning tests. Overall these findings suggest that technological tools are not motivating in themselves, but that they can provide a more motivating context in the service of learning. Along this line, Ritella et al. highlighted the importance of considering new technologies as an innovative teaching tool to be used, however, in a theoretical framework. They analyzed students' perceptions and lasting memories of a course delivered in a mixed modality and studied the transfer of skills and knowledge over 10 years. The course was based on the model of constructive and collaborative participation (CCP) (Cucchiara et al., 2014) and included activities involving each student individually or in interaction with other students. The students reported vivid memories of the teaching methods and contents of the course, but of most interest, they stated that they use the soft skills acquired during the course in their current work activities. These findings reaffirm the importance of designing educational experiences that allow students to build knowledge through individual and collaborative activities that can take advantage of technological innovations.

The last issue referred to novel technologies in teaching, offering insights about teachers' cognitions of online educational paradigms. Chen et al.'s work (Chen et al.), through semi-structured interviews with teachers, provided evidence that using interactive spherical video-based virtual reality (ISV-VR) might be effective to improve descriptive composition writing in L1, in secondary school. The authors underlined that applying VR had an impact not only on students' skills and motivation but also on teachers' perspectives, which from “teacher-centered” became “student-centered.” This contribution highlighted the need for teachers to increase their skills in integrating new technologies in teaching-learning processes. Rüth and Kaspar's work (Rüth and Kaspar) offers interesting clues about the use of commercial videogames in high-school courses. They proposed the use of a videogame on the evolution by natural selection in a 10th grade biology course and the use of a videogame on the First World War in a 12th grade course on history. Such activities increased motivation and contributed to share experiences not only on the subject contents but also on coding skills, promoting discussions on the way to improve the videogames. The authors provided evidence that videogames at school can be used as “objects-to-think-with.” Gao and Zhang offered an interesting framework to integrate teachers' representations of the use of ICT in teaching English as a Foreign Language (EFL) in Chinese universities during the pandemic breakout. Moving from Koehler and Mishra (2005) model (TPACK) that integrates the perception of technology use within teachers' cognition (Borg, 2015), technological knowledge (TK) interacts with pedagogical knowledge (PK), and content knowledge (CK) in teachers' representations of on-line teaching EFL. Qualitative analysis of in-depth interviews with three EFL teachers showed that teachers are aware of the positive and negative aspects of the use of ICT in teaching English; they agree that the digital skills, acquired during the pandemic emergency, might be used to integrate traditional classroom teaching with online activities.

In conclusion, the contributions point out new research directions to inform educational practices and bridge the gap between technology innovation and educational methodologies, offering new perspectives of development for researchers and stakeholders.

## Author Contributions

MV worked on the initial alignment of ideas and worked on subsequent editing. BC, DS, ML, and DT worked on specific sections. ML completed final editing. All authors contributed to the article and approved the submitted version.

## Funding

This publication was supported by the CARIPLO Foundation [Grant 2017-NAZ-0131, Tecnologie Innovative per il Benessere e l'Integrazione Scolastica (IBIS) (New technologies for education and their impact on students' well-being and inclusion)].

## Conflict of Interest

The authors declare that the research was conducted in the absence of any commercial or financial relationships that could be construed as a potential conflict of interest.

## Publisher's Note

All claims expressed in this article are solely those of the authors and do not necessarily represent those of their affiliated organizations, or those of the publisher, the editors and the reviewers. Any product that may be evaluated in this article, or claim that may be made by its manufacturer, is not guaranteed or endorsed by the publisher.
